# Polymicrobial Infection of the Cornea Due to Contact Lens Wear

**DOI:** 10.4274/tjo.03779

**Published:** 2016-04-05

**Authors:** Selçuk Sızmaz, Sibel Bingöllü, Elif Erdem, Filiz Kibar, Soner Koltaş, Meltem Yağmur, Reha Ersöz

**Affiliations:** 1 Çukurova University Faculty of Medicine, Department of Ophthalmology, Adana, Turkey; 2 Çukurova University Faculty of Medicine Central Laboratory, Department of Microbiology, Adana, Turkey; 3 Çukurova University Faculty of Medicine, Department of Medical Parasitology, Adana, Turkey

**Keywords:** Acanthamoeba, Alcaligenes, keratitis, Pseudomonas

## Abstract

A 38-year-old male presented with pain and redness in his left eye. He had a history of wearing contact lenses. His ophthalmic examination revealed a large corneal ulcer with surrounding infiltrate. Cultures were isolated from the contact lenses, lens solutions, storage cases, and conjunctivae of both eyes and also corneal scrapings of the left eye. Fortified vancomycin and amikacin drops were started hourly. Culture results of conjunctivae of each eye and left cornea were positive for Pseudomonas aeruginosa; cultures from the contact lenses, lens solution and storage case of both eyes revealed Pseudomonas aeruginosa and Alcaligenes xylosoxidans. Polymerase chain reaction of the corneal scraping was positive for Acanthameoba. The topical antibiotics were changed with ones that both bacteria were sensitive to and anti-amoebic therapy was added. The patient had two recurrences following initial presentation despite intensive therapy. Keratitis occurred due to multiple pathogens; the relapsing course despite adequate therapy is potentially associated with this polymicrobial etiology.

## INTRODUCTION

Infectious keratitis is a sight-threatening complication of contact lens (CL) wear. Most types of lenses have been reported to be associated with infectious keratitis. For a favorable outcome, it is essential to identify the causative agent. To date, several microbiological agents including bacteria, fungi and protozoa have been reported. It is known that microorganisms can reside on lenses and lens storage cases, and CL solutions also act as reservoirs for microbial growth. The latter is more strongly correlated with keratitis.^1,2^

The aim of this case report is to describe a case of polymicrobial keratitis due to CL wear. The causative agents identified were Pseudomonas aeruginosa, Alcaligenes xylosoxidans, and Acanthameoba. Ali and Reddy^3^ reported a case of bilateral polymicrobial keratitis in which Pseudomonas aeruginosa, Alcaligenes spp., and Flavobacterium meningosepticum were involved. To the best of our knowledge, this is the first published case of CL-related polymicrobial keratitis with the aforementioned organisms.

## CASE REPORT

A 38-year-old otherwise healthy male presented to our outpatient cornea and CL clinic with complaints of pain, redness, photophobia and blurred vision in his left eye since the previous day. He had been using soft CLs for a period of time and had not had a follow-up ophthalmologic examination during this period. He could not give clear information about the commercially available brands of CL and CL solution he used. He was wearing daily CLs and replacing them monthly. He had slept with his lenses for two consecutive nights prior to these complaints, as he had frequently done. He placed the lenses in a cleaning solution when he removed them. He reported that he routinely showered while wearing his lenses, indicating a history of exposure to possibly contaminated water. He had used dexamethasone/netilmicin combination and ketorolac drops, which were not prescribed by a physician.

**Figure 1:**
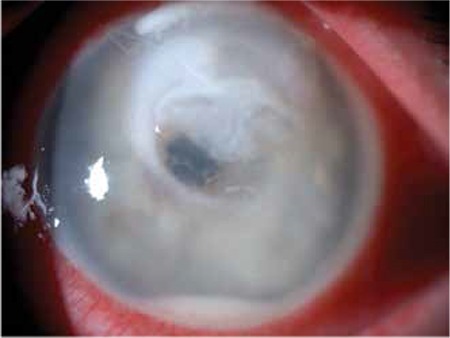
The initial presentation of the patient with large corneal ulcer and surrounding infiltrate involving the center and the upper half of the cornea, stromal thinning, and hypopyon in the left eye

In his ophthalmologic examination, the right eye had 20/20 best-corrected visual acuity and both anterior and posterior segment findings were normal. His visual acuity in the left eye was counting fingers. Slit-lamp biomicroscopy revealed ciliary injection, large corneal ulcer (4x4 mm in size), and surrounding infiltrates involving the center and the upper half of the cornea, stromal thinning, and hypopyon (Figure 1). The fundus could not be visualized.

The patient was diagnosed with a CL-related corneal ulcer in the left eye and was hospitalized. Corneal scraping, cultures and Gram staining of the cornea and the conjunctiva were obtained. Right and left CLs, both lens storage cases, and two separate CL solutions were also sent for culture and Gram staining. Corneal scrapings were also examined by both polymerase chain reaction (PCR) and real time-PCR for the parasite Acanthameoba. Fortified vancomycin (50 mg/ml) and fortified amikacin (50 mg/ml) drops were started hourly. Additionally, cyclopentolate and artificial tears were prescribed.

Right and left conjunctival and left corneal cultures were positive for Pseudomonas aeruginosa. The remaining cultures revealed the following: left CL, Pseudomonas aeruginosa and Alcaligenes xylosoxidans; right CL, Pseudomonas aeruginosa; both CL storage cases, Pseudomonas aeruginosa and Alcaligenes xylosoxidans. The two microorganisms were sensitive to amikacin, piperacillin, and tazobactam. Hence, fortified vancomycin was replaced with fortified piperacillin (7 mg/ml). As corneal, left CL and left storage case cultures revealed positive PCR test for Acanthamoeba, hourly chlorhexidine (0.2 mg/ml) and propamidine isethionate (1 mg/ml) drops were added to the aforementioned regimen. On the 10th day of hospitalization, the hypopyon disappeared, the ulcer healed and corneal haze diminished. The patient’s visual acuity improved to 20/400 and he was discharged; the hourly drops were diminished to every three hours.

**Figure 2:**
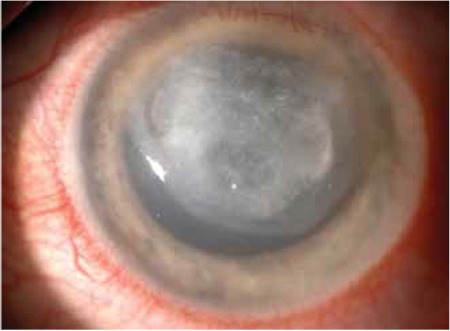
The left eye 3 weeks after initial presentation. Note, there is a crescent-shaped stromal thinning at the nasal edge of the ulcer

**Figure 3:**
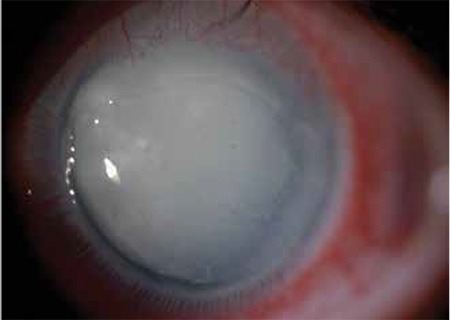
The large persistent epithelial defect involving the majority of the left cornea at final presentation

In the third week of follow-up, the patient presented with diminished vision (counting fingers) and pain, although he was under close follow-up and receiving the drops every three hours. The ulcer was enlarged (7x6 mm) and there was a crescent-shaped stromal thinning at the nasal edge of the ulcer (Figure 2). Because these recent findings were thought to have occurred secondary to extensive use of the fortified antibiotics including chlorhexidine and propamidine, they were decreased to QID. Stromal thinning and the epithelial defect healed. After discharge the patient was lost to follow-up. In the 3rd month after initial presentation, he was admitted with total corneal opacity and a persistent epithelial defect in which a majority of the cornea was involved with stromal scarring resembling a persistent ulcer (Figure 3).

### Microbiology Tests

All the samples were inoculated onto Columbia agar with 5% sheep blood, MacConkey agar, and chocolate agar with polyvitex for the aerobic bacterial culture and incubated at 37 °C for 24-48 hours. Specimens were inoculated onto Schaedler agar with 5% sheep blood and chocolate agar with polyvitex for the anaerobic bacteria culture and incubated in a jar including an AnaeroGen kit (BD GasPak anaerobe container system, Maryland, USA) at 37 °C for a week.

Achromobacter xylosoxidans and Pseudomonas aeruginosa were identified using Vitek-2 GN (gram-negative) identification cards (Vitek 2 System, Biomerieux, USA). Antimicrobial susceptibility testing of these isolates was done using the Vitek-2 system. The criteria of the Clinical and Laboratory Standards Institute were used to interpret the antimicrobial resistance patterns of the isolates.

## DISCUSSION

All three causative organisms that were obtained from the current case are very well-known pathogens for CL-related keratitis, of which Pseudomonas aeruginosa is the most common.^1,2,3,4,5,6^ Acanthamoeba spp. are free-living protozoan parasites and are particularly reported to be associated with poor hygienic conditions.^7^ On the other hand, despite its relatively low prevalence Alcaligenes (formerly Achromobacter) xylosoxidans is an important cause of infection in CL wearers. Alcaligenes spp. are gram-negative, aerobic bacilli; although rare, they may cause keratitis, particularly in compromised corneas.^5,6^

Poor hygiene conditions enhance susceptibility to keratitis in CL wearers.^7^ Overnight CL wear is a common compromising factor for corneal infections.^8^ It was clear that our patient did not take good care of his hygiene and did not have any follow-up visits with an ophthalmologist. He was also unable to provide information regarding the brands CL and cleaning solution he was using. He had a prior history of two nights sleeping with CLs, which he reported to be actually longer. Moreover, he had a history of regular and uncontrolled topical corticosteroid use. As a result of these factors the patient became susceptible to a polymicrobial keratitis with destructive organisms. Also, the relapsing nature of the infection can be attributed to the polymicrobial etiology due to poor hygiene.

Pseudomonas and Alcaligenes were involved in both eyes of our patient, whereas Acanthamoeba was isolated from the left eye. As the patient had no sign of keratitis in his right eye, Acanthamoeba, which was isolated only from the left eye, is strongly considered to be the causative agent. On the other hand, we believe that the role of the other two organisms must be discussed in terms of their contribution to the clinical outcome. It might be considered that the polymicrobial nature of the infection could affect its course, resulting in high recurrence rates and poor visual acuity. We also considered possible toxic effects of intensive topical therapy including fortified antibiotics; hence, the drops were tapered in the second hospitalization, which was three weeks after the first one. Our patient had no problem with compliance to therapy during follow-up; however, Alcaligenes is reported to be particularly associated with frequently recurrent keratitis.^9^

One must always consider accompanying topical anesthetic abuse in cases with severe pain. Topical anesthetic abuse itself is known to cause superinfections with poor outcome.^10,11^

Silicone-hydrogel CLs are known to be associated with significantly lower Pseudomonas aeruginosa binding than conventional extended-wear soft CLs.^4^ However, as we had no knowledge about the type of the CL our patient was wearing, we are not able to establish a relationship between the CL type and enhanced microbial binding.

Sharma et al.^12^ stressed the risk of co-infection in their report, in which the patient did well following administration of propamidine isethionate 0.1%, chlorhexidine 0.02%, and polymyxine B eye drops in addition to previous anti-pseudomonal therapy. Immediately following presentation, we started fortified vancomycin and fortified amikacin. Both Pseudomonas and Alcaligenes were found to be sensitive to amikacin, piperacillin, and tazobactam. Hence, vancomycin was changed with piperacillin. As Acanthamoeba was identified by PCR, we started anti-amoebic therapy as well. Besides the polymicrobial nature, we believe that the intense topical therapy which would be expected to alter epithelial healing might also have played a role in the persistent course of the infection. As we tapered the topical regimen, the ulcer started to heal. Our plan was to administer topical therapy for an appropriate period of time, but the patient was lost to follow-up and later presented with recurrence.

## CONCLUSION

To the very best of our knowledge this is the first report concerning these three organisms. Pseudomonas and Acanthamoeba are well known causes of CL keratitis, whereas because of the relative difficulty in its identification, the role of Alcaligenes might be underestimated.^6^ Moreover, Alcaligenes spp. should be kept in mind in such persistent keratitis cases.

## Ethics

Informed Consent: Obtained.

Peer-review: Externally peer-reviewed.
